# Effects of Support Surface and Shooting Action on Muscle Activity of Trunk Muscles in Ice Hockey Players: A Cross-Sectional Study

**DOI:** 10.3390/jcm14062090

**Published:** 2025-03-19

**Authors:** Seongmin Oh, Taewoong Jeong, Yijung Chung

**Affiliations:** 1 Department of Physical Therapy, The Graduate School, Sahmyook University, Seoul 01795, Republic of Korea; 2 Department of Rehabilitation Medicine, Soonchunhyang University Bucheon Hospital, Bucheon 14584, Republic of Korea; 3 Department of Physical Therapy, College of Health and Welfare, Sahmyook University, Seoul 01795, Republic of Korea

**Keywords:** athlete, ice hockey, electromyography, muscle activity, unstable surface

## Abstract

**Background:** This study aimed to investigate the effect of different support surfaces on trunk muscle activity during slap shots and wrist shots in ice hockey players by analyzing muscle activation patterns across varying conditions. **Methods:** A total of 22 healthy male collegiate ice hockey players participated in this study. Ice hockey players were assessed for muscle activity in their trunk muscles (rectus abdominis, external oblique, internal oblique, and erector spinae). Each player performed a slap shot and wrist shot on solid ground, a slap shot and wrist shot on ice, and a skating slap shot and wrist shot on ice. Data from a 3 s interval, excluding the first and last second of the 5 s measurement period, were used for analysis. All electromyography signals were normalized using maximal voluntary isometric contraction. **Results:** Significant differences were found in all muscles except for the external oblique, depending on the support surface and shooting method. (*p* < 0.05). The muscle activity of the rectus abdominis was significantly greater for the slap shot and wrist shot on ice than for the slap shot and wrist shot on the ground, and the slap shot and skating slap shot on ice than for the wrist shot on the ground (*p* < 0.05). The internal oblique was significantly higher for slap shots and wrist shots on ice than for slap shots and wrist shots on the ground (*p* < 0.05). The erector spinae was significantly greater for the skating slap shot and wrist shot on ice than for the wrist shot on the ground, and the skating slap shot on ice was significantly greater than the skating wrist shot on ice (*p* < 0.05). **Conclusions:** To enhance the shooting efficiency of ice hockey players competing on ice, exercises on unstable surfaces and targeted trunk muscle training are considered to be effective.

## 1. Introduction

The support surface is crucial for maintaining balance by generating ground reaction forces to correct unbalanced movements [1]. To maintain balance on an unstable surface, more muscles are engaged for a longer duration compared to a stable surface [2]. When exercising on an unstable surface, similar effects can be achieved with lower intensity compared to performing the same movements on a stable surface [3]. Unlike a stable surface, the direction in which the body sways differs on an unstable surface, leading to a different pattern of muscle cooperation to maintain balance [4]. On a stable surface, when an athlete exerts force, the body’s center of mass moves in the opposite direction of the force applied, whereas on an unstable surface, it moves in the direction of the applied force [5]. Previous studies have shown that unstable surfaces generally increase trunk muscle activity, particularly in global muscles such as the rectus abdominis and external obliques, while local muscle activation, including the transversus abdominis and lumbar multifidus, varies depending on the exercise [6]. It remains unclear how trunk muscle activation differs specifically during sport-specific movements, such as an ice hockey shot, performed on stable and unstable surfaces.

In ice hockey, shooting is an essential skill, and the two most common types of shots are the slap shot and the wrist shot [7]. Players strive to maximize their goal-scoring potential by increasing the speed and accuracy of these shots, which are predominantly used in games. Numerous studies aim to identify the equipment, movement patterns, and biomechanics that enable the fastest shot execution [8].

The primary goal of the slap shot is to shoot the puck at maximum speed [9]. The slap shot generates a maximum puck speed of over 30 m/s for both forwards and defensemen [10] and is characterized by a faster puck speed compared to other shots performed by ice hockey players, such as the wrist, snap, and sweep shots [11]. This powerful technique involves striking the ice before the puck with the blade of the stick to generate speed [12]. In ice hockey, forwards use the slap shot 26% of the time, while defensemen use it 54% of the time [7].

The wrist shot is the most accurate type of shot performed to score in ice hockey games [13]. Because the wrist shot involves less swing than the slap shot, it can be executed more quickly [14]. When greater accuracy is required, the puck is propelled at speeds exceeding 20 m/s with a snap or push motion by swinging the stick forward [10]. The wrist shot is a technique that involves pre-loading the stick (causing it to bend forcefully against the ice) and maintaining contact with the puck from the start [15]. Depending on the player’s position, the wrist shot accounts for 23–37% of all shots [13]. Ice hockey players decide which shot to perform based on the situation; if a powerful shot is needed, they use the slap shot, swinging the stick to hit the puck with maximum force. If time is limited, a quick wrist shot is more ideal [15].

Robbins et al. stated that strong trunk rotation during an ice hockey shot increases both puck and blade speed [16]. The trunk significantly affects an athlete’s ability to generate and transfer force through the limbs [17] and plays a crucial role in maintaining stability and balance while performing movements with the limbs [18]. To effectively execute trunk rotation in sports like tennis strokes or golf swings, the strength of the trunk muscles is also necessary to generate speed and force during rotation. These trunk muscles are highly active during the acceleration phase of trunk rotation [19]. Donald et al. argued that trunk muscles are crucial for the performance of athletes in sports requiring significant trunk rotation [20]. Robbins et al. investigated the relationship between puck and blade speed and trunk rotation during ice hockey shots [16]. Bežák and Přidal studied male ice hockey players to examine the relationship between maximum puck speed in slap shots and wrist shots and the player’s upper body strength, finding a significant association between shot speed and upper body strength and power [21]. Donald et al. investigated the electromyographic activity of the rectus abdominis, external oblique, and erector spinae muscles during slap shots and wrist shots performed by female ice hockey players on the ground. Their study reported greater trunk muscle activity during the slap shot compared to the wrist shot [20].

Previous studies have compared lower limb muscle activity during exercise and skating in ice hockey players [22,23], investigated factors influencing shot speed [16,21], and examined trunk muscle activity during shots executed on solid surfaces [20]. However, although the ice surface is the fundamental condition for ice hockey matches and training, research in the biomechanics of ice hockey shots on ice has been limited due to the difficulties in data collection on ice.

The hypotheses of this study are as follows. First, the muscle activity of specific trunk muscles will differ depending on the type of surface. Based on previous studies, it is hypothesized that the activity of the global muscles (external oblique, internal oblique, and rectus abdominis) will significantly increase on an unstable surface, as these muscles are more involved in maintaining balance and generating force [6]. In contrast, the erector spinae may show a moderate increase due to its stabilizing role in postural adjustments. Second, muscle activity of these trunk muscles will also differ depending on the type of shot. Specifically, it is expected that slap shots, which require greater force and dynamic movement, will result in higher activation of the global muscles (external oblique, internal oblique, and rectus abdominis) compared to wrist shots, which involve relatively less force and dynamic demand. The erector spinae is hypothesized to show consistent activation across both shot types due to its primary role in maintaining spinal stability. Third, there will be an interaction between the type of surface and the type of shot in determining trunk muscle activity. For instance, performing a slap shot on an unstable surface is expected to lead to the highest activation of the global muscles due to the combined demands of dynamic force generation and balance maintenance.

Therefore, this study aims to measure trunk muscle activity in ice hockey players while performing slap shots and wrist shots on both solid ground and ice surfaces. The goal is to investigate how different support surfaces affect trunk muscle activity during these shots.

## 2. Materials and Methods

### 2.1. Participants

In this study, ice hockey players enrolled at K University were recruited. The inclusion criteria were as follows: physically and mentally healthy players, without any cardiovascular conditions, able to independently perform slap shots and wrist shots, and capable of following the researcher’s instructions appropriately. The exclusion criteria were as follows: players with injuries that could potentially affect the research results. A total of 22 male ice hockey players were recruited, but one player was excluded due to limited access to ice rink time. Ultimately, the study was conducted with 21 ice hockey players, consisting of 13 forwards and 8 defensemen.

The required sample size was calculated using G-Power software (ver. 3.1.9.7; Heinrich Heine University, Dusseldorf, Germany). An F test (repeated measures ANOVA) was employed with an effect size of 0.4, an alpha level of 0.05, and a statistical power of 0.8. The calculation resulted in a required sample size of 18 participants. Considering a dropout rate of 20%, the total sample size was set to 22 participants.

Participants who voluntarily agreed to take part in the study after being informed about the procedures, objectives, and potential use of the results signed a consent form. The study was approved by the Institutional Review Board of Sahmyook University (Approval No. SYU 2024-04-007). Registered with the Clinical Research Information Service, this study adheres to the World Health Organization International Clinical Trials Registry Platform (WHO-ICTRP) guidelines (registration number: KCT0010093).

### 2.2. Study Procedure

This study employed a cross-sectional study design to compare trunk muscle activity across six different experimental conditions in ice hockey players. A total of 22 healthy male collegiate ice hockey players in their 20 s who met the selection criteria were recruited. However, due to logistical constraints, one player was excluded, resulting in 21 participants in the final study. The general characteristics of the participants, including age, height, weight, age at which they started playing, years of experience, and dominant side, were assessed.

**Figure 1:**
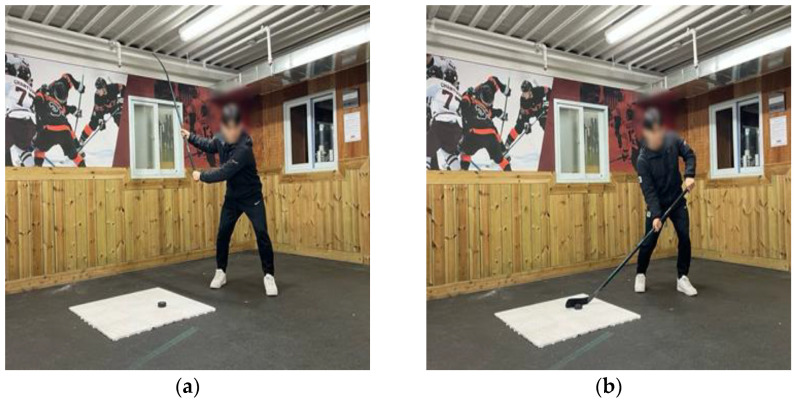
Starting position on solid ground: (**a**) slap shot; (**b**) wrist shot.

**Figure 2:**
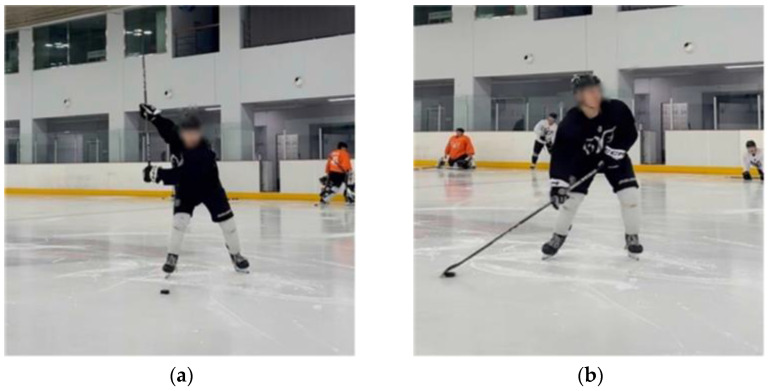
Starting position on ice: (**a**) slap shot; (**b**) wrist shot.

**Figure 3:**
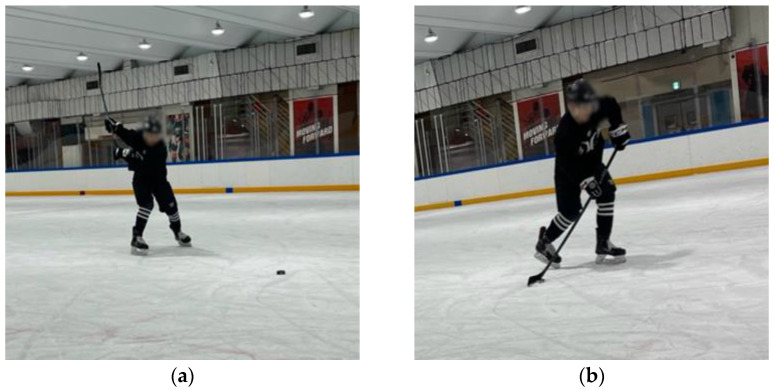
Starting position on ice while skating: (**a**) slap shot; (**b**) wrist shot.

The six experimental conditions were as follows: slap shots and wrist shots on solid ground (Figure 1), slap shots and wrist shots on ice (Figure 2), and skating slap shots and wrist shots on ice (Figure 3). The slap shot involves initially raising the stick blade backward and can be described in six phases: backswing, downswing, pre-loading, loading, release, and follow-through. In contrast, the wrist shot begins with the stick blade in contact with the puck and typically consists of four or five phases: draw back (optional), blade positioning, loading, pushing, and follow-through. In this study, the ice skating task was performed by initiating skating from the blue line and executing the shot, whereas the standing task was conducted from a stationary position, similar to a typical ground condition, with the shot being taken from the puck’s set position. The puck was placed at the midpoint between the two face-off circles located within the end zone of the ice rink to ensure consistency across trials. Each action was performed in a random order, and each movement was repeated three times to obtain the average muscle activity. To minimize muscle fatigue from the movements, a 3 min rest was allowed between each action. The starting position for the slap shot involved holding the stick with both hands at a distance of 0.4 to 0.6 m. The trunk was flexed 45° and rotated 70° in the direction opposite to the puck [10]. After assuming this position, the puck was shot using a swing, and then the player returned to the starting position [24]. The starting position for the wrist shot involved holding the stick with both hands at a distance of 0.15 to 0.30 m [10]. The shot was performed from a comfortable stance with the shoulders perpendicular to the goal [25].

### 2.3. Outcome Measures

In this study, surface electromyography (Ultium EMG System, Noraxon, AZ, USA) was used to measure muscle activity. The sampling rate of the electromyography (EMG) signals was set to 2000 Hz, and the frequency bandwidth was set between 20 and 500 Hz. First, the hair at the electrode attachment sites was removed using a disposable razor. Then, the electrode sites were cleaned with an alcohol swab to remove any foreign substances or oil from the skin. After this preparation, the electrodes were attached, and measurements were taken. The placement of the electrodes for the study was as follows: for the rectus abdominis, the electrodes were positioned 2 cm lateral and 1 cm superior to the umbilicus; for the internal oblique, the electrodes were placed on the upper part midway between the anterior superior iliac spine and the inguinal ligament; for the external oblique, the electrodes were attached 15 cm lateral to the umbilicus, between the lower rib cage and the anterior superior iliac spine; and for the erector spinae, the electrodes were positioned 2 cm lateral from the spinous process of the second lumbar vertebra [26]. The measured EMG signals were processed using full-wave rectification with MR3 software (MyoResearch, Noraxon, AZ, USA).

The electrodes used were general-purpose Ag/AgCl electrodes containing a moisture gel component. When attaching the electrodes, a distance of 2 cm between them was maintained. After properly positioning the electrodes, the maximal voluntary isometric contraction (MVIC) of the muscles was measured. Based on the manual muscle test posture, the rectus abdominis, internal oblique, and external oblique were measured individually in the supine position, while the erector spinae was measured in the prone position [27]. The normalization method involved using the average EMG signal recorded over 3 s, excluding the first and last 1 s of a 5 s measurement taken with a start command. This average was used to normalize the MVIC.

### 2.4. Statistical Analysis

All data processing and statistical analyses were conducted using PASW version 22.0 (SPSS Inc., Chicago, IL, USA). Normality tests were performed on the data, and the general characteristics of the subjects were analyzed using descriptive statistics. To examine the differences in trunk muscle activity among ice hockey players based on support surfaces and shooting postures, a one-way repeated analysis of variance (ANOVA) was conducted. Post hoc analysis was performed using the least significant difference (LSD) test, and the significance level for all statistical analyses was set at 0.05.

## 3. Results

Initially, 22 participants were recruited. However, due to logistical constraints, one participant was excluded, resulting in a final total of 21 participants. The study included 21 male ice hockey players (n = 21) with an average age of 21.61 ± 1.28 years. The participants consisted of 13 forwards and 8 defensemen. They began training at an average age of 9.42 ± 2.42 years and had an average training experience of 12.19 ± 2.53 years. Their average height and weight were 178.95 ± 4.6 cm and 76.33 ± 3.9 kg, respectively. Regarding stick handling preference, 9 players used the right side as their dominant stick side, while 12 used the left side. The muscle activity of the dominant side trunk muscles of ice hockey players, according to support surface and shooting posture, is shown in Table 1.

### 3.1. Muscle Activity of the Rectus Abdominis

The muscle activity of the rectus abdominis was significantly greater during slap shots and skating slap shots on ice compared to wrist shots on solid ground (*p* < 0.05). Additionally, the muscle activity was significantly greater during slap shots and wrist shots on ice, as well as skating slap shots and wrist shots on ice, compared to slap shots on solid ground (*p* < 0.05).

### 3.2. Muscle Activity of the External Oblique

The muscle activity of the external oblique did not differ significantly between slap shots and wrist shots on ice, and skating slap shots and wrist shots on ice, compared to slap shots and wrist shots on solid ground.

### 3.3. Muscle Activity of the Internal Oblique

The muscle activity of the internal oblique was significantly greater during slap shots and wrist shots on ice, as well as skating slap shots and wrist shots on ice, compared to slap shots and wrist shots on solid ground (*p* < 0.05).

### 3.4. Muscle Activity of the Erector Spinae

The muscle activity of the erector spinae was significantly greater during slap shots and wrist shots on ice, as well as skating slap shots and wrist shots on ice, compared to wrist shots on solid ground (*p* < 0.05). Additionally, muscle activity during skating slap shots on ice was significantly greater than during skating wrist shots on ice (*p* < 0.05).

## 4. Discussion

This study demonstrated that trunk muscle activity in ice hockey players was significantly greater during shots performed on ice compared to solid ground, emphasizing the increased stabilization demands of unstable surfaces. Additionally, slap shots showed higher trunk muscle activation than wrist shots, highlighting the greater rotational and stabilization requirements involved in slap shots. These findings provide important insights into the biomechanical demands of ice hockey shooting techniques under varying surface conditions.

In ice hockey, shooting is an essential skill, with the two most common types of shots being the slap shot and the wrist shot [7]. Bežák and Přidal used slap shots and wrist shots to investigate the relationship between maximum puck speed and upper body strength and power in ice hockey players [21], while Kurz et al. employed these shots to examine the impact of a specialized hockey composite test on shot performance, specifically puck speed and accuracy, in ice hockey players [28]. Donald et al. used slap shots and wrist shots in a study that examined trunk muscle activity in female hockey players during shooting [20]. Therefore, in this study, slap shots and wrist shots were selected to investigate the impact of shooting posture on trunk muscle activity in ice hockey players.

Robbins et al. stated that strong trunk rotation during an ice hockey shot increases puck and blade speed [16], while Torén noted that the erector spinae plays a crucial role in stabilizing the trunk during rotational movements [29]. Skinkle et al. noted that the trunk significantly influences an athlete’s ability to generate and transfer force to the limbs [17], and Rasouli et al. stated that the trunk muscles consist of the rectus abdominis, internal obliques, and external obliques [30]. Therefore, in this study, the muscle activity of the rectus abdominis, internal obliques, external obliques, and erector spinae was measured in ice hockey players performing slap shots and wrist shots on both solid ground and ice.

Lam et al. stated that performing exercises on an unstable surface can yield similar effects to those achieved on a stable surface, even when the exercises are performed at a lower intensity [3]. Additionally, Kim et al. found that standing on one leg on an unstable round foam roller activates the trunk muscles more than performing the same exercise on a stable floor [31]. This finding aligns with the results of this study, where muscle activity in the trunk was significantly greater during shots on ice compared to shots on solid ground. Additionally, Snarr and Esco measured trunk muscle activity during plank exercises on different surfaces and found that activity was significantly higher on unstable surfaces compared to stable ones [32]. As with previous studies, it has been shown that unstable surfaces increase muscle activation levels and enhance coordinated muscle activation patterns, thus emphasizing the neural control of movement [33].

In this study, the muscle activity of the rectus abdominis during wrist shots on ice and skating wrist shots on ice did not show a significant difference compared to wrist shots on solid ground. Bežák and Přidal investigated the correlation between upper body strength and slap shots and wrist shots in ice hockey players and found that upper body strength attributes contribute more to puck speed when executing wrist shots compared to slap shots [21]. Choi and Lee highlighted that the angular displacement and coordination patterns of the trunk and upper limbs (shoulder-elbow, elbow-wrist connections) play a crucial role in ice hockey shots, and the coordination patterns between the trunk-shoulder, shoulder-elbow, and elbow-wrist are essential for the successful execution of a shot [34]. The lack of significant difference in muscle activity between wrist shots on ice and skating wrist shots compared to wrist shots on solid ground in this study suggests that during a wrist shot, the movement of the wrist contributes more to accuracy and rapid execution, while the movement of the upper limbs is more involved than the trunk.

In this study, the muscle activity of the erector spinae did not show significant differences between wrist shots and slap shots on ice, as well as skating wrist shots and slap shots on ice, compared to slap shots on solid ground. When performing a slap shot, the trunk bends at 45-degree angles in the direction opposite to the puck and rotates at 70-degree angles before swinging the stick [24]. Hass et al. reported that the range of motion of the trunk was reduced in the rotational plane when exercising on unstable surfaces, attributing this to the increased need for muscle stabilization on unstable surfaces, which can be seen as a functional limit of the muscles [35]. This result suggests that when performing a slap shot on an unstable surface, the increased demand for muscle stabilization imposes greater strain on the muscles responsible for trunk rotation, thereby reducing the trunk’s rotational range. Hamaoui et al. suggested that higher muscle tension leads to reduced stability of the body, which can cause a decrease in the range of motion due to muscle stiffness on unstable surfaces [36]. Additionally, Dean et al. stated that as the maximum reach distance increases, there is an increase in both foot support and muscle activity in the legs [37]. On unstable surfaces, if the leg muscles reach their functional limit earlier in the process of stabilizing from the beginning, the support capacity is limited, which can restrict the maximum rotational distance. In this study, the muscle activity of the erector spinae did not show significant differences between slap shots on ice and skating slap shots compared to slap shots on solid ground. This is likely because, when performing a slap shot on ice, the erector spinae requires greater stabilization of the trunk, which can restrict the range of trunk rotation. On solid ground, however, the erector spinae was less active in stabilizing the trunk, allowing for a greater range of trunk rotation and thus leading to no significant difference in muscle activity.

The findings of this study highlight the importance of trunk stabilization for optimizing shooting performance in ice hockey players. The significantly higher trunk muscle activity observed during shots on ice compared to solid ground suggests that incorporating unstable surface training into conditioning programs can enhance players’ ability to stabilize their trunk during dynamic on-ice movements. For example, exercises such as single-leg balance drills on unstable platforms or foam rollers can be included to mimic the stabilization demands of ice hockey. Moreover, the greater trunk muscle activity during slap shots compared to wrist shots indicates that training programs should focus on developing rotational strength and coordination for slap shots. Plyometric exercises, medicine ball rotational throws, and resistance band rotations can be used to target trunk rotational strength and power.

These practical recommendations can help coaches and trainers design sport-specific conditioning programs that improve shot efficiency, accuracy, and overall performance in competitive settings. Additionally, integrating these exercises may help reduce the risk of injuries by improving trunk stability and movement control during high-intensity on-ice actions.

This study had a limitation in that the sample size was restricted to players from a single university and consisted only of males, which limits the generalizability of the findings to the broader population of ice hockey players. Additionally, muscle activity was measured only on one side of the body. Given the asymmetrical and complex nature of rotational movements, future research should consider measuring muscle activity on both sides to provide a more comprehensive understanding of muscle activation patterns.

## 5. Conclusions

This study demonstrated that trunk muscle activity in ice hockey players was greater during shots on ice compared to solid ground, with higher activation observed during slap shots than wrist shots. These findings highlight the importance of trunk stabilization and suggest that incorporating unstable surface training and targeted trunk muscle exercises can enhance shooting performance on ice.

## Figures and Tables

**Table 1:** Muscle activation according to support surface and shooting posture.

	Rectus Abdominis	External Oblique	Internal Oblique	Erector Spinae
GS (%MVIC)	16.14 ± 6.19	39.72 ± 21	27.42 ± 17.67	59.38 ± 16.61
GW (%MVIC)	18.67 ± 11.37	31.45 ± 16.52	28.21 ± 17.3	47.25 ± 19.15
IS (%MVIC)	29.11 ± 15.14 ^a,b^	38.47 ± 11.04 ^a,b^	38.08 ± 14.62	71.82 ± 29.99 ^b^
IW (%MVIC)	25.62 ± 13.76 ^a^	37.09 ± 14.24 ^a,b^	36.5 ± 17.35	59.45 ± 18.18 ^b^
ISS (%MVIC)	28.61 ± 17.23 ^a,b^	42.94 ± 15.26 ^a,b^	43.69 ± 19.19	73.64 ± 27.01 ^b,c^
ISW (%MVIC)	23.78 ± 11.33 ^a^	35.14 ± 14.1 ^a,b^	40.01 ± 16.83	60.73 ± 20.97 ^b^
F	3.953	1.363	4.958	13.351
*p*	0.003	0.259	0.001	0.003

Values are mean ± standard deviation. GS: ground standing slap shot; GW: ground standing wrist shot; IS: ice standing slap shot; IW: ice standing wrist shot; ISS: ice skating slap shot; ISW: ice skating wrist shot; MVIC: maximal voluntary isometric contraction. ^a^ Conditions that showed a significant difference from GS. ^b^ Conditions that showed a significant difference from GW. ^c^ Conditions that showed a significant difference from ISW.

## Data Availability

The original contributions presented in this study are included in the article. Further inquiries can be directed to the corresponding author(s).
